# Terrorist Attacks Against COVID-19-Related Targets during the Pandemic Year 2020: A Review of 165 Incidents in the Global Terrorism Database

**DOI:** 10.1017/S1049023X22002394

**Published:** 2023-02

**Authors:** Harald De Cauwer, Dennis G. Barten, Derrick Tin, Luc J. Mortelmans, Bart Lesaffre, Francis Somville, Gregory R. Ciottone

**Affiliations:** 1. Department of Neurology, Sint-Dimpna Regional Hospital, Geel, Belgium; Faculty of Medicine and Health Sciences, University of Antwerp, Wilrijk, Belgium; 2. Department of Emergency Medicine, VieCuri Medical Center, Venlo, the Netherlands; 3. Department of Emergency Medicine, Beth Israel Deaconess Medical Center, Boston, Massachusetts; Harvard Medical School, Boston, Massachusetts; 4. Center for Research and Education in Emergency Care, University of Leuven, Leuven; REGEDIM, Free University Brussels, Brussels; Department of Emergency Medicine, ZNA Camp Stuivenberg, Antwerp, Belgium; 5. Department of Emergency Medicine, AZ Sint Jan, Bruges/Oostende, Belgium and HoWest, Bruges, Belgium; 6. Department of Emergency Medicine, Sint-Dimpna Regional Hospital, Geel, Belgium; Faculty of Medicine and Health Sciences. University of Antwerp, Wilrijk, Belgium; Faculty of Medicine, University of Leuven, Leuven, Belgium; CREEC (Center for Research and Education in Emergency Care). University of Leuven, Leuven, Belgium; 7. Director, BIDMC Disaster Medicine, Beth Israel Deaconess Medical Center; Associate Professor, Harvard Medical School, Boston, Massachusetts, USA

**Keywords:** conspiracy theory, Counter-Terrorism Medicine, COVID-19, pandemic, terrorism, vaccination

## Abstract

**Background:**

The coronavirus disease 2019 (COVID-19) pandemic enabled a situational type of terrorism with mixed racist, anti-government, anti-science, anti-5G, and conspiracy theorist backgrounds and motives.

**Objective:**

The objective of this study was to identify and characterize all documented COVID-19-related terrorist attacks reported to the Global Terrorism Database (GTD) in 2020.

**Methods:**

The GTD was searched for all COVID-19-related terrorist attacks (aimed at patients, health care workers, and at all actors involved in pandemic containment response) that occurred world-wide in 2020. Analyses were performed on temporal factors, location, target type, attack and weapon type, attacker type, and number of casualties or hostages. Ambiguous incidents were excluded if there was doubt about whether they were exclusively acts of terrorism.

**Results:**

In total, 165 terrorist attacks were identified. With 50% of incidents, Western Europe was the most heavily hit region of the world. Nonetheless, most victims were listed in Southeast Asia (19 fatalities and seven injured). The most frequent but least lethal attack type concerned arson attacks against 5G telephone masts (105 incidents [60.9%] with only one injured). Armed assaults accounted for most fatalities, followed by assassinations. Incendiary and firearms were the most devastating weapon types.

**Conclusion:**

This analysis of the GTD, which identified 165 COVID-19-related terrorist attacks in 2020, demonstrates that the COVID-19 pandemic truly resulted in new threats for COVID-19 patients, aid workers, hospitals, and testing and quarantine centers. It is anticipated that vaccination centers have become a new target of COVID-19-related terrorism in 2021 and 2022.

## Introduction

The coronavirus disease 2019 (COVID-19) pandemic started in China at the end of 2019. The Chinese government warned the World Health Organization (WHO; Geneva, Switzerland) that a new virus had been detected; however, before the health authorities fully collected information on the virus’ characteristics, it spread within China, and subsequently around the world, despite the early containment and management measures aimed at reducing the exponential growth of the pandemic through social distancing, isolation, and other non-pharmaceutical measures.^1–3^

The first COVID-19-related terrorist attack mentioned in the Global Terrorism Database (GTD) occurred in Hong Kong on January 27, 2020 where a small homemade bomb was used to target a local hospital.^4^

Later in 2020, other COVID-19-related attacks were observed, spreading globally, following a similar trajectory as the virus. Fear of the “new Chinese virus” and disinformation around it seemed to not only have negative psychological impacts, but also to influence non-state actors in committing terrorist attacks.^4,5^

Some (violent) far-left, far-right, and Islamist extremist groups incorporated COVID-19 into their narratives.^6^

The objective of this study is to identify and characterize all documented COVID-19-related terrorist attacks in the GTD during the pandemic year 2020.

## Methods

A database search of the GTD was performed on July 11, 2022 by using the Preferred Reporting Items for Systematic Reviews and Meta-Analyses (PRISMA) standard.^7^ The GTD is an open-source database containing over 200,000 global terrorism incidents that occurred in the period from 1970-2020. The GTD is maintained by the National Consortium for the Study of Terrorism and Responses to Terrorism (START) at the University of Maryland (College Park, Maryland USA) and is part of the US Department of Homeland Security (Washington, DC USA) Center of Excellence.^8,9^

The GTD defines a terrorist attack as follows: *“the threatened or actual use of illegal force and violence by a non-state actor to attain a political, economic, religious, or social goal through fear, coercion, or intimidation.”* ^9^ To be considered for inclusion in the GTD, the following three attributes must all be present:

1. The incident must be intentional;
2. The incident must entail some level of violence or immediate threat of violence; and
3. The perpetrators of the incidents must be subnational actors.

Additionally, to be included in the database, two out of three of the following criteria must be present:

1. The act must be aimed at attaining a political, economic, religious, or social goal;
2. There must be evidence of an intention to coerce, intimidate, or convey some other message to a larger audience than the immediate victims; and/or
3. The action must be outside the context of legitimate warfare activities.

An extensive description of the origin and the data collection methodology can be found in the GTD codebook, which is available on the START website.^9^ The full dataset of the GTD was searched for COVID-19-related terrorist attacks.

The GTD codebook does not provide a specific definition of COVID-19-related incidents.^9^ For this study, the following procedure was applied. Terrorist attacks aimed at patients and caregivers, and at pharmaceutical and health care facilities, were assessed. To be eligible for inclusion, these attacks had to be COVID-19-related, both pro- or contra-COVID-19 treatment/isolation. Incidents targeting the management of the pandemic (by testing, isolation, containment, or lockdown measures) by politicians, governmental institutions, police, military, and health care workers were also included.

The following search terms were applied in the database: “vaccin” and “vaccine,” “COVID,” “Corona,” “virus,” and “viral.” Duplicates were excluded. Each specific attack of coordinated incidents is listed separately in the GTD.

Cases in which there was insufficient information to determine whether it was COVID-19-related were further explored using reviews of gray literature. If information remained insufficient, the cases were excluded. Incidents coded as “Doubt Terrorism Proper” were also excluded. These are incidents in which there was doubt if they were exclusively terrorism-related.^9^

Data collected per incident included temporal and spatial factors; location (country, world region); intended target; attack and weapon type; perpetrator type; and number of casualties.

Each entry was reviewed manually by the lead researcher for inclusion or exclusion based on the incident description. A second author (LM) reviewed each entry, as well as the excluded incidents. In case of doubt or discrepancies, a third author (DB) advised on the final decision. All collected data were exported into Excel spreadsheets (Microsoft Corporation; Redmond, Washington USA) and analyzed descriptively.

## Results

**Figure 1.**
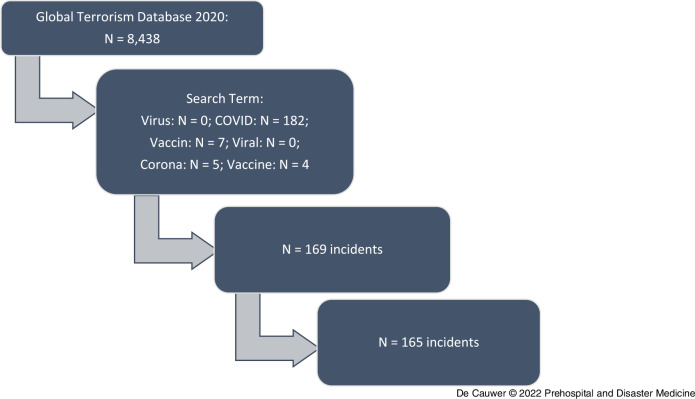
PRISMA Diagram. Step 1: *Identification* of all registered incidents in the GTD. Step 2: *Screening* for incidents with search terms. Step 3: *Eligibility* – N = 29 incidents excluded because of polio vaccine-related (N = 9), doubt of terrorism (N = 1), other not COVID-19-related (N = 19). Step 4: *Final Inclusion* with N = 4 duplicates excluded.

In 2020, the GTD contained 8,438 incidents, of which 165 fulfilled the inclusion criteria (Figure 1). The attacks occurred in 34 countries and on six continents.

### Incidents per Country and Region

The United Kingdom (n = 31; 18.8%), the Netherlands (n = 30; 18.2%), the Philippines (n = 14; 8.5%), and New Zealand (n = 12; 7.3%) were the most commonly affected countries (Table 1).

**Table 1.** Incidents and Victim Numbers by Country, Listed in the GTD 2020

Country	Incidents	Fatalities	Injured
Afghanistan	1	0	0
Australia	3	0	1
Belgium	2	0	0
Bolivia	7	0	0
Brazil	1	1	0
Cameroon	1	0	3
Canada	8	0	0
China	4	0	0
Colombia	3	5	4
Cyprus	6	0	0
Ecuador	1	0	0
Finland	1	0	0
France	3	0	0
Germany	4	0	0
Greece	2	0	0
India	2	0	0
Indonesia	1	1	1
Iran	1	2	1
Ireland	3	0	0
Italy	1	0	0
Kenya	1	0	0
Myanmar	2	1	1
Netherlands	30	0	0
New Zealand	12	0	0
Pakistan	1	0	1
Philippines	14	17	5
Poland	2	0	0
Russia	1	0	0
Somalia	3	1	10
Turkey	1	2	1
United Kingdom	31	0	1
United States	7	0	0
Yemen	4	1	1
Zimbabwe	1	1	1
**Total**	**165**	**32**	**31**

**Figure 2.**
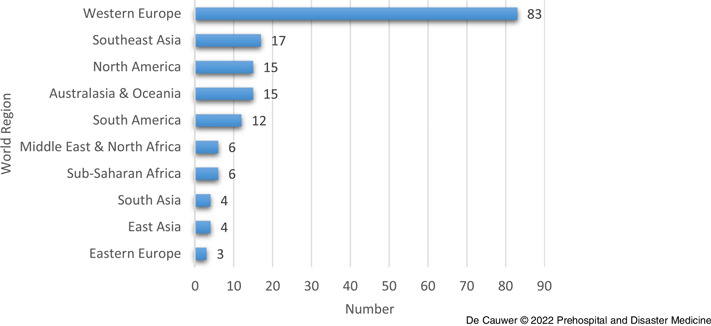
Distribution of COVID-19-Related Incidents in 2020 by World Region.

With 83 (50.3%) out of 165 attacks, Western Europe was the world region with the highest number of incidents (Figure 2). Southeast Asia ranked second with 17 (10.3%) attacks, followed by North America and Australasia & Oceania, both with 15 (9.1%) attacks.

### Number of Victims per Country and World Region

**Figure 3.**
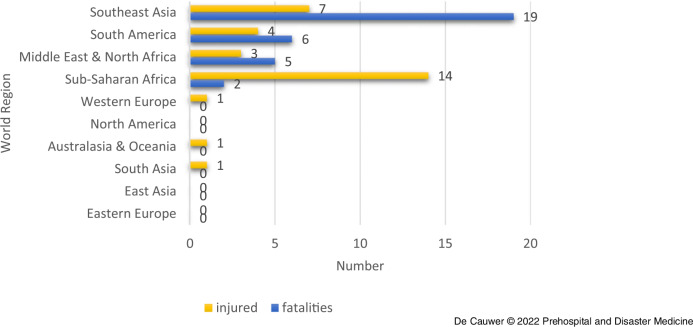
Fatalities and Injured by World Region.

In total, 32 confirmed fatalities were registered in the GTD. A total of 31 people were injured (Table 1 and Figure 3). There was no clear relation between the number of incidents and the number of victims.

The Philippines (n = 17; 53.1%) and Colombia (n = 5; 15.6%) accounted for the most fatalities.

The regions with the most fatalities were Southeast Asia (n = 19; 59.4%), South America (n = 6; 18.7%), and the Middle East & North Africa (n = 5; 15.6%); Figure 3. In Western Europe, South Asia, North America, East Asia, Eastern Europe, and Australia & Oceania, no fatalities were to deplore.

Most injured people were counted in Sub-Saharan Africa (n = 14; 45.1%), Southeast Asia (n = 7; 22.6%), and South America (n = 4; 12.9%).

When assailants thought to be from the New People’s Army (NPA), Philippines attempted to attack a COVID-19 quarantine checkpoint, security forces responded and killed four assailants. This was the only incident mentioning fatalities among perpetrators.^10^

### Perpetrator Group Type and Number of Casualties per Perpetrator Group Type

Various perpetrator groups and related motives were mentioned in the GTD (Table 2). However, in some of them, data were missing or perpetrators were not known (n = 22; 13.3%). In some perpetrators, the motives and any relation to existing terrorist factions could not be demonstrated, while in other attacks, no one claimed responsibility.

**Table 2.** Number of Incidents by Perpetrator Group Type and Fatalities and People Injured by these Factions

Perpetrator Type	No. Incidents	People Killed (n)	People Injured (n)
Conspiracy Theory Extremists	113	0	1
Unknown	22	14	21
Left-Wing and Communist	11	9	2
Separatists	8	5	6
Islamic Jihadi	5	4	1
Anti-Asian	2	0	0
Immigrants	2	0	0
Far-Right	1	0	0
Anti-Government Extremists	1	0	0
**Total**	**165**	**32**	**31**

For those perpetrators for whom the GTD presented enough information, (suspected) conspiracy theory extremists were predominant, with N = 113 (68.5%) incidents (Table 2). Other, less-frequent perpetrators factions consisted of left-wing extremists and communist factions, separatists, and Islamic groups. A unique group included immigrants in a refugee camp in Moria, Greece who set fire in response to forced COVID-19 testing and quarantine.

The GTD only listed two incidents in the United States targeting members of the Nepalese Gurkha community related to racially motivated violence in the wake of COVID-19 fears.

Another remarkable incident consisted of a far-right inspired active member of the Canadian armed forces driving his vehicle through the gates of the residence of Justin Trudeau, the Prime Minister of Canada, at Rideau Hall in Ottawa, Ontario, Canada. Sources stated that he posted QAnon and other far-right conspiracy theories online.

There was no correlation between the number of incidents and the medical impact.

In fact, left-wing extremists and communist factions accounted for only 11 incidents, but nine fatalities and two injured people (Table 2). Separatist factions were in the second place, with five fatalities and six injured, followed by Islamic factions, with four fatalities and one injured. For the majority of the victims, however, a specific perpetrator group could not be assigned.

Only four lone actor attacks were registered in the GTD: the Canadian assailant and a Belgian unaffiliated individual who threw a petrol bomb at the fence of the Belgian Parliament building in Brussels, Belgium. After being taken in custody, the man stated that the attack was in protest of the government’s handling of the COVID-19 pandemic.^11^

In Michigan, United States, a lone actor wanted to attack both a police station and Standish hospital where he intended to “disrupt the power to the hospital, unlock the doors, and release patients under COVID-19 quarantine.”^12^ Authorities believed that he attempted to steal the helicopter to aid in his attack on the police station and hospital.

Another assailant derailed a locomotive in an attempt to have it crash into the United States Navy Hospital Ship Mercy in California, United States. The man attempted to “wake people up” about the presence of the hospital ship because he was suspicious that the ship may have been docked to spread COVID-19 or to facilitate a government takeover.^13^

### Target Type

The most frequent attack type concerned arson attacks against 5G telephone masts (105 incidents [60.9%] resulting in one person with injuries and no fatalities). In most attacks, no group claimed responsibility for the incident. In all of these cases, the GTD states that “authorities suspected that the incident may have been carried out in relation to conspiracy theory regarding the link between 5G radio waves and the COVID-19 pandemic” (Table 3).^14^

**Table 3.** Number of Incidents per Target Group Type and Fatalities and People Injured

Target type	No. Incidents	People Killed (n)	People Injured (n)
5G	105	0	1
Aid Workers	7	7	5
Government	7	3	0
Quarantine/Isolation Center	6	10	1
Military	5	4	1
Hospital	5	0	1
Police	4	1	13
Border Control	4	0	3
Preacher	2	1	1
Testing Center	2	0	1
Citizens	1	3	4
Medical Doctor	1	2	0
Journalist	1	1	0
**Total**	**165**	**32**	**31**

As such, these incidents were not targeting patients, health care, or the pharmaceutical industry. However, they were aimed at governmental pandemic response and met the definition for inclusion in this series.

Aid workers (mostly primary care) and police and military were other frequent targets. Hospitals (n = 6), medical doctors, and governmental personnel who were managing the COVID-19 pandemic or worked at testing centers of quarantine facilities were less frequently involved in the incidents (Table 3).

In two incidents, places of worship/preachers were attacked because services were still on-going, and assailants were driven by fear of spreading COVID-19.

In six incidents, the motives were driven by fear that current pandemic management was insufficient or foreigners/immigrants/churches were at risk of spreading the disease. This contrasts with the majority of attacks, which targeted lockdown/testing/isolation/government measures (n = 148).

### Weapon Type

Figures for attack type show that armed assaults accounted for most fatalities, followed by assassinations, and data for weapon type show that incendiary and firearms were the most devastating (Table 4).

**Table 4.** Number of Incidents, Fatalities, and Injuries per Attack Type and Weapon Type

Attack type	Incidents (n)	People Killed (n)	People Injured (n)	Weapon Type	Incidents (n)	People Killed (n)	People Injured (n)
Infrastructure Attack	120	0	1	Incendiary	119	16	25
Armed Assault	20	24	11	Firearms	23	13	4
Hostage Taking	9	0	5	Mixed	9	1	1
Bombing	8	0	12	Explosives	7	2	0
Assassination	6	6	1	Unknown	5	0	1
Unknown	2	2	1	Melee	1	0	0
				Vehicle	1	0	0
**Total**	**165**	**32**	**31**		**165**	**32**	**31**

This series lists 45 coordinated attacks on the same day, the majority being incendiaries targeting telecommunication (5G).

## Discussion

Ever since the Middle Ages, pandemics coexist with pandemic-related terrorism. Therefore, COVID-19-related terrorist incidents were predictable.^15–17^ Throughout history, assailants aimed their terrorist activities at health care workers, patients, foreigners, minorities, and governments. Several factors involved in hostility and violence during pandemics were previously identified: fear (ie, of income loss, losing jobs, of the disease, or vaccination); religious and sociocultural motives; governments acting against health care workers and scientists; ethnic considerations, racism, and hatred; doom thinking and conspiracy theories; anti-government and anti-foreigner motives; and war zone and regional conflicts.^15^

As mentioned, the first COVID-19-related attacks were observed in Hong Kong and mainland-China in January 2020.^4^ These initial attacks were thought to result from the fear of contracting and spreading the virus, and targeted infected patients and isolation facilities. These incidents, listed in the GTD as terrorist incidents, were not related to anti-government riots and demonstrations against the political influences of mainland-China in the formerly British Hong Kong territories. Six Hong Kong-based attacks were excluded because the relation with the pandemic could not be established. For the similar reasons, five attacks by the New People’s Army in The Philippines were excluded from this series. Although there are gray in these events, mixed motives could be the case.

The American President Trump calling the new coronavirus “the Chinese virus,” followed by the global spread of the pandemic, with Italy becoming the first bridgehead for the siege of Europe, are thought to have played a role in racism-driven terrorism: both Asian and Italian people endured racist narratives of doom-thinking and conspiracy theory believers.^4^ In this series of terrorist incidents listed in the GTD, however, no attacks targeting these specific populations were included. The anti-science and anti-health care narrative, mostly mixed with anti-government and racist motives, resulted in some lone actors planning attacks against Jewish people, hospitals, and isolation and COVID-19 testing centers to free patients from “unreliable” health care services.^4,17^

The majority of incidents were arson attacks against 5G communications masts, but resulted in only one injury. As most of these events were not accounted for, with the assailants remaining unknown in most cases, it can only be inferred that conspiracy thinking and perhaps anti-science sentiment are the underlying motive for these attacks. These incidents occurred in many countries and often on the same day or time period, raising concern that they were part of a concerted action, perhaps through social media. Another possibility is that some of these incidents were carried out by so-called copy-cats.

Once the COVID-19 vaccines became available in late 2021, large-scale vaccination campaigns were started. Incidents resulting from anti-vaccination efforts can be assessed in a follow-up study as soon as the GTD is updated with the 2021 and 2022 incidents. It is known that historically, vaccinators have been a target for terrorists.^16,18^ The COVID-19-vaccination is the latest on a long list of disputed health care campaigns. In 2020, nine polio vaccination campaigns were also targeted, according to the GTD.^19^

As scientists and the health care community are threatened by “anti-vaxxers,” as shown in two recent surveys, a call for the incorporation of counter-terrorism measures into all national and international (eg, WHO) anti-pandemic guidelines and strategies is needed.^20,21^

The UN Institute for Training and Research (UNITAR; Geneva, Switzerland) also calls for increased protection and target hardening of hospitals. Hospitals remain one of the few remaining soft targets while pandemic measures restrict the movement of people, thus also assailants, in public space.^22^

During the COVID-19 pandemic, a disinformation campaign via social media posts praised the COVID-19 response in Russia and spread fake news about outcome figures in other countries. This campaign consisted of stigmatization of certain population groups, and was launched in an attempt to undermine the Western governmental pandemic response.^23^

Preventive measures against anti-science and anti-health care narratives (including fact checks of narratives launched by unreliable foreign state-sponsored groups) are probably more effective than a late response. A proactive push against false information seems to be more effective than trying to persuade people who have already adopted it.

## Limitations

The GTD is the most comprehensive, up-to-date, open access and reliable database of terrorist incidents.^8,9^ However, the database, and therefore this study, is subject to several limitations.

The GTD relies on media publications for their information with only high-quality sources used, creating a possible selection bias.^8,9^

Casualty numbers conflict across sources. Following the GTD protocol, the most recent reliable estimates are reported and used in this study.

Attempted but unsuccessful attacks are included in the GTD. However, threats, conspiracies, or the planning of attacks are not. The perpetrators literally had to be “out the door” to be included as an incident.

Additionally, state terrorism is of growing importance, but it is not included in the GTD.^8,9^

## Conclusion

This analysis of the GTD, which identified 165 COVID-19-related terrorist attacks in 2020, demonstrates that the COVID-19 pandemic indeed resulted in new threats for COVID-19 patients, aid workers, hospitals, and testing and quarantine centers. It is anticipated that vaccination centers have also become a new target of COVID-19-related terrorism in 2021 and 2022.
